# Increased Incidence of Antimicrobial-Resistant Nontyphoidal *Salmonella* Infections, United States, 2004–2016

**DOI:** 10.3201/eid2706.204486

**Published:** 2021-06

**Authors:** Felicita Medalla, Weidong Gu, Cindy R. Friedman, Michael Judd, Jason Folster, Patricia M. Griffin, Robert M. Hoekstra

**Affiliations:** Centers for Disease Control and Prevention, Atlanta, Georgia, USA

**Keywords:** *Salmonella*, nontyphoidal, antimicrobial resistance, antibiotic resistance, drug resistance, ceftriaxone, ciprofloxacin, ampicillin, incidence, resistance trends, foodborne diseases, bacteria, United States, enteric infections

## Abstract

*Salmonella* is a major cause of foodborne illness in the United States, and antimicrobial-resistant strains pose a serious threat to public health. We used Bayesian hierarchical models of culture-confirmed infections during 2004–2016 from 2 Centers for Disease Control and Prevention surveillance systems to estimate changes in the national incidence of resistant nontyphoidal *Salmonella* infections. Extrapolating to the United States population and accounting for unreported infections, we estimated a 40% increase in the annual incidence of infections with clinically important resistance (resistance to ampicillin or ceftriaxone or nonsusceptibility to ciprofloxacin) during 2015–2016 (≈222,000 infections) compared with 2004–2008 (≈159,000 infections). Changes in the incidence of resistance varied by serotype. Serotypes I 4,[5],12:i:- and Enteritidis were responsible for two thirds of the increased incidence of clinically important resistance during 2015–2016. Ciprofloxacin-nonsusceptible infections accounted for more than half of the increase. These estimates can help in setting targets and priorities for prevention.

Nontyphoidal *Salmonella* infections cause an estimated 1.2 million illnesses, 23,000 hospitalizations, and 450 deaths each year in the United States (*1*). Although most infections result in self-limited illness, antimicrobial treatment is recommended for patients with severe infection or at high risk for complications (*2*). Antimicrobial-resistant *Salmonella* infections can cause adverse clinical outcomes, including increased rates of hospitalization, bloodstream infection, other invasive illnesses, and death (*3*–*7*).

Nontyphoidal *Salmonella* infections can be acquired during international travel, from contaminated food and water, through animal contact, and from environmental sources (e.g., wetlands and irrigation water) (*8*–*13*). Antimicrobial-resistant infections have been linked to various food and animal sources (*3*,*14*,*15*). In 2015 and previous years, 5 commonly isolated serotypes (Enteritidis, Typhimurium, Newport, I 4,[5],12:i:- and Heidelberg) accounted for more than half of antimicrobial-resistant *Salmonella* infections in the United States (*16*–*20*). The distribution of antimicrobial-resistant infections caused by some of these common serotypes varied by region (*21*,*22*).

In a previous study, we found that an estimated annual average of 6,200 culture-confirmed infections were resistant to ceftriaxone or ampicillin or nonsusceptible to ciprofloxacin during 2004–2014 (*20*). For the study described in this article, we used the same modeling approach and data sources to estimate changes in incidence. We estimated the contribution of the 5 major serotypes to changes in incidence and describe differences by geographic region. We extrapolated findings to the United States population to provide estimates to help set targets and priorities for reducing antimicrobial resistance among nontyphoidal *Salmonella*.

## Methods

### Laboratory-Based Enteric Disease Surveillance

Public health laboratories in 50 states and many local health departments receive human *Salmonella* isolates from clinical laboratories and report serotype information to the Centers for Disease Control and Prevention (CDC) through Laboratory-Based Enteric Disease Surveillance (LEDS) (*17*). We excluded serotypes Typhi, Paratyphi A, Paratyphi B (tartrate-negative), and Paratyphi C, which account for <1% of *Salmonella* infections in the United States, and whose only known reservoir are humans (*2*,*16*,*17*,*23*). In this article, we use the term *Salmonella* to refer to nontyphoidal *Salmonella*.

### National Antimicrobial Resistance Monitoring System

The National Antimicrobial Resistance Monitoring System (NARMS) is a collaboration among CDC, the US Food and Drug Administration, the US Department of Agriculture, and state and local health departments to monitor resistance among enteric bacteria isolated from humans, retail meat, and food animals (*16*,*24*). Public health laboratories in 50 state and 4 local health departments submit every 20th *Salmonella* isolate received from clinical laboratories to CDC for antimicrobial drug susceptibility testing (*16*,*19*,*24*).

During 2004–2016, CDC tested *Salmonella* isolates for susceptibility to agents representing 8–9 antimicrobial classes: aminoglycosides, β-lactam/β-lactamase inhibitors, cephems, macrolides (tested since 2011), penici­­llins, quinolones, folate pathway inhibitors, phenicols, and tetracyclines (*16*). MICs were determined by broth microdilution with Sensititer (ThermoFisher, https://www.thermofisher.com) and interpreted using criteria from the Clinical and Laboratory Standards Institute (CLSI) when available (*7*,*16*). Using CLSI criteria, we defined ceftriaxone resistance as MIC >4 µg/mL, ampicillin resistance as MIC >32 µg/mL, and nonsusceptibility to ciprofloxacin as MIC >0.12 µg/mL. The ciprofloxacin definition includes both resistant and intermediate CLSI categories because *Salmonella* infections with intermediate susceptibility to ciprofloxacin have been associated with poor treatment outcomes (*6*,*7*,*16*).

### Resistance Categories

We defined clinically important resistance as resistance to ceftriaxone, nonsusceptibility to ciprofloxacin, or resistance to ampicillin on the basis of the following criteria: third-generation cephalosporins (e.g., ceftriaxone) and fluoroquinolones (e.g., ciprofloxacin) are used for empiric treatment of severe infections; fluoroquinolones are not recommended for children; and ampicillin is useful for susceptible infections (*2*). We defined and ranked from highest to lowest 3 mutually exclusive categories of clinically important resistance (Appendix Figure 1) (*20*): ceftriaxone/ampicillin resistance (because all ceftriaxone-resistant isolates are ampicillin-resistant); ciprofloxacin nonsusceptibility (nonsusceptible to ciprofloxacin but susceptible to ceftriaxone); and ampicillin-only resistance (ampicillin-resistant but susceptible to ceftriaxone and ciprofloxacin). We included ciprofloxacin-nonsusceptible isolates that were ceftriaxone-resistant in the ceftriaxone/ampicillin category because they are of greatest public health and treatment concern. Many isolates in each category had resistance to other agents. We defined multidrug resistance as resistance to >3 classes of antimicrobial agents (*16*,*19*).

### Bayesian Hierarchical Model to Estimate Changes

We used 2004–2016 data from LEDS, NARMS, and the US Census Bureau as input in the models (*16*,*17*,*25*). For LEDS, we used the number of culture-confirmed infections by state and year (state-year). We combined serotyped isolates other than Enteritidis, Typhimurium, Newport, I 4,[*5*],12:i:-, and Heidelberg into an “other” category. We assigned unserotyped and partially serotyped isolates from each state into the 6 serotype categories (Enteritidis, Typhimurium, Newport, I 4,[*5*],12:i:-, Heidelberg, and other) on the basis of the average proportion of serotyped isolates in each category from 2004–2016. For NARMS, we used resistance proportions among fully serotyped isolates per state-year. We used US Census population data for each state-year to express incidence per 100,000 persons per year (*25*).

A similar Bayesian hierarchical model approach was used from a previous study to estimate the incidence of resistant infections (*20*,*26*). However, we found a Poisson model for LEDS data better captured the uncertainty of *Salmonella* incidence observed at the state-year level instead of the normal distribution used in our previous study (*20*). The model incorporated the random effects of state, year, and state-year interaction to borrow strength from contiguous states and previous years (*20*,*26*–*28*). Alaska and Hawaii were excluded because they are not adjacent to any state; the District of Columbia was also excluded, which began submitting isolates to NARMS in 2008 (*16*,*19*,*20*). We used an approach similar to our previous study to make adjustments for data from Florida, which reported low numbers of isolates compared with its 6 closest states (*17*,*18*,*20*).

**Figure 1:**
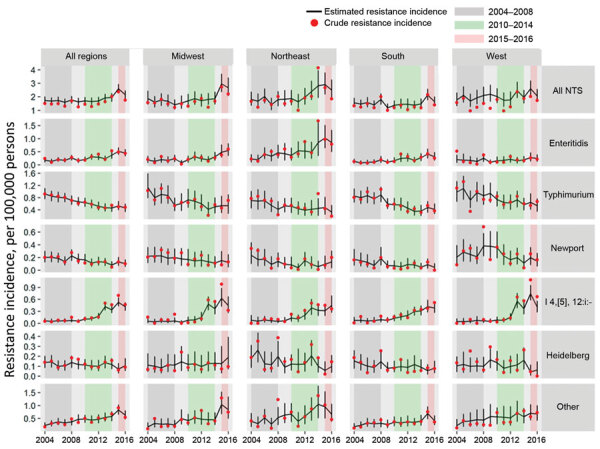
Estimated annual incidence of culture-confirmed nontyphoidal *Salmonella* infections with any clinically important resistance, by serotype and region, United States, 2004–2016. Estimated changes in resistance incidence (mean and 95% credible intervals of the posterior differences per 100,000 persons per year) were derived using Bayesian hierarchical models. Crude resistance incidence rates were derived by multiplying infection incidence and resistance proportion for state-year. Any clinically important resistance was defined as resistant to ceftriaxone, resistant to ampicillin, or ciprofloxacin nonsusceptible. The “other” category comprised serotypes other than Enteritidis, Typhimurium, Newport, I 4,[*5*],12:i:-, and Heidelberg. US Census regions were used to define 4 geographic regions. NTS, all nontyphoidal *Salmonella* serotypes.

We applied the models to generate estimates (referred to as posterior estimates) for *Salmonella* infection incidence rates, resistance proportions, and resistant infection incidence rates (referred to as resistance incidence) by state-year for each of the 6 serotype categories by using Markov chain Monte Carlo simulations (*20*,*26*–*28*). For each serotype category, we estimated resistance incidence for overall clinically important resistance, the 3 mutually exclusive categories of clinically important resistance, and multidrug resistance. For all *Salmonella*, we calculated overall estimates by summing estimates across the 6 serotype categories. We calculated state-year resistance incidence estimates per 100,000 persons per year as estimated incidence for state-year × estimated resistance proportion for state-year. For estimation of resistance incidence by geographic region, we used the 4 US Census region categories (Midwest, Northeast, South, and West) and aggregated posterior estimates of resistance incidence by year for all states in each region (*25*). For each resistance category, we calculated mean estimates and 95% credible intervals (CrIs) from posterior estimates and mean crude rates by year for the 48 states and those stratified by serotype and region categories (Figure 1; Appendix Figures 2–5) for an overall side-by-side comparison (*20*,*25*,*26*).

To assess changes in resistance incidence, we compared the mean resistance incidence from 2015–2016 with that from two 5-year reference periods during 2004–2016: 2004–2008 and 2010–2014. These reference periods are consistent with those used in NARMS annual reports to assess changes in resistance percentages (*16*). All 50 states have participated in NARMS since 2003; the 2004–2008 period is the early years of nationwide participation and the 2010–2014 period is the recent past. For each resistance and serotype category, we calculated the difference between the posterior estimates of resistance incidence for 2015–2016 and those for each year in the 5-year reference periods for each region to obtain the mean difference and the 95% CrIs. We did not assume homogeneous rates across multiple years using this approach. For all *Salmonella*, we calculated the change in resistance incidence, which represents the net change (increase or decrease), for each resistance category, by summing the estimated changes derived for the 6 serotype categories. We describe statistically significant changes (i.e., in which the 95% CrIs do not include 0).

### Extrapolating to the US Population

We multiplied the mean estimates of culture-confirmed infections by 29, which is the estimated number of total infections for every culture-confirmed infection in the general population, to estimate the total number of resistant infections for each period and changes in total resistant per 100,000 persons per year during 2015–2016 compared with the reference periods for each resistance category (*1*). We used the average 2015–2016 population estimates for the 50 states (322 million) to extrapolate to the US population (*25*).

## Results

During 2004–2016, public health laboratories of state and participating local health departments in the 48 contiguous states reported 539,862 culture-confirmed *Salmonella* infections to LEDS (Appendix Table 1). Among the isolates from these infections, 89% were serotyped; the most common were Enteritidis (20%), Typhimurium (16%), Newport (11%), I 4,[*5*],12:i:- (4%), and Heidelberg (4%). Public health laboratories in the 48 states submitted 28,265 isolates to NARMS. Of these isolates, 98% were serotyped; the most common were Enteritidis (19%), Typhimurium (16%), Newport (11%), I 4,[*5*],12:i:- (4%), and Heidelberg (4%).

### Clinically Important Resistance and Multidrug Resistance

During 2004–2016, clinically important resistance was detected in 3,546 (12.5%) of 28,265 isolates (Table 1; Appendix Figure 1). Ampicillin-only resistance was detected in 1,857 (6.6%) isolates, ciprofloxacin nonsusceptibility in 854 (3.0%), and ceftriaxone/ampicillin resistance in 835 (3.0%). Only 78 (0.3%) isolates were resistant to ceftriaxone and nonsusceptible to ciprofloxacin; these isolates were included in the 835 categorized as ceftriaxone/ampicillin-resistant. Most (>90%) ciprofloxacin-nonsusceptible isolates had MICs within the intermediate range, 0.12–0.5 (Table 1; Appendix Figure 6).

**Table 1:** Number and percentage of antimicrobial-resistant nontyphoidal *Salmonella* isolates, by serotype and resistance category, United States, 2004–2016*

Resistance category	No. (%) isolates							
	Enteritidis, n = 5,206	Typhimurium, n = 4,404	Newport, n = 3,140	I 4,[5],12:i:-, n = 1,158	Heidelberg, n = 974	Other fully serotyped, n = 12,878	Not fully serotyped, n = 505	Total nontyphoidal *Salmonella*, N = 28,265
Any clinically important resistance†	548 (10.5)	1,197 (27.2)	284 (9.0)	389 (33.6)	240 (24.6)	843 (6.5)	45 (8.9)	3,546 (12.5)
Multidrug resistance‡	114 (2.2)	1,178 (26.7)	271 (8.6)	382 (33.0)	204 (20.9)	727 (5.6)	36 (7.1)	2,912 (10.3)
Amp-only§	152 (2.9)	897 (20.4)	30 (1.0)	319 (27.5)	120 (12.3)	311 (2.4)	28 (5.5)	1,857 (6.6)
Cef/Amp§¶	15 (0.3)	212 (4.8)	237 (7.5)	39 (3.4)	116 (11.9)	212 (1.6)	4 (0.8)	835 (3.0)
Cipro§#	381 (7.3)	88 (2.0)	17 (0.5)	31 (2.7)	4 (0.4)	320 (2.5)	13 (2.6)	854 (3.0)

*Amp-only, resistant to ampicillin (MIC >32 µg/mL) but susceptible to ceftriaxone and ciprofloxacin; Cef/Amp, resistant to ceftriaxone (MIC >4 µg/mL) and ampicillin; Cipro, nonsusceptible to ciprofloxacin (MIC >0.12 µg/mL) but susceptible to ceftriaxone; NTS, nontyphoidal *Salmonella,* which includes isolates serotyped as Enteritidis, Typhimurium, Newport, I 4,[*5*],12:i:-, and Heidelberg, isolates serotyped as other than those 5, and those not fully serotyped. †Includes any of the 3 clinically important resistance patterns (i.e., resistant to ceftriaxone, resistant to ampicillin, or nonsusceptible to ciprofloxacin). Isolates might have resistance to other agents tested. ‡Resistant to >3 classes of antimicrobial agents. §Amp-only, Cef/Amp, and Cipro are mutually exclusive categories of clinically important resistance. ¶Of the 835 isolates with Cef/Amp resistance, 78 (0.3% of all nontyphoidal *Salmonella* isolates) were nonsusceptible to ciprofloxacin. Of the 78 isolates, 71 (91%) had ciprofloxacin MICs within the intermediate range (i.e., 0.12–0.5) (Appendix Figure 6). These 78 isolates were not included in the Cipro category. #Of the 854 isolates, 785 (92%) had ciprofloxacin MICs within the intermediate range (Appendix Figure 6).

Of the 28,265 isolates, 2,912 (10.3%) were multidrug resistant (MDR). Of these, 2,633 (90%) had clinically important resistance, which accounted for 74% of the 3,546 isolates with clinically important resistance.

### Incidence by Year and Region, 2004–2016

For each resistance category, the trend lines were smoother with model-derived annual estimates of resistance incidence compared with crude rates, particularly when stratified by serotype and region (Figure 1; Appendix Figures 2–5). Crude rates tended to be lower than model-derived estimates because many state-year resistance proportions used in calculating crude rates were 0 because of small sample sizes, whereas the model tended to pull estimates away from 0. Overall, most crude rates were within model-derived 95% CrIs.

### Resistance Incidence, 2015–2016

During 2015–2016, the mean annual incidence was 2.38 (95% CrI 1.93–2.86)/100,000 persons for clinically important resistant infections and 1.83 (95% CrI 1.45–2.25)/100,000 persons for MDR infections (Table 2). The 5 major serotypes accounted for 69% of infections with clinically important resistance and 66% with multidrug resistance.

**Table 2:** Estimated incidence and changes in the incidence of antimicrobial-resistant culture-confirmed nontyphoidal *Salmonella* infections, by resistance category, United States, 2015–2016 versus 2004–2008 and 2010–2014*

Resistance category	Mean (95% CrI)					
					Change in resistance incidence, per 100,000 persons per year‡	
	Resistance incidence, per 100,000 persons per year†				2015–2016 vs. 2004–2008	2015–2016 vs. 2010–2014
	2015–2016	2004–2008	2010–2014			
Any clinically important resistance§	2.38 (1.93–2.86)	1.70 (1.44–1.98)	1.78 (1.46–2.15)		**0.68 (0.13 to 1.24)‡**	0.60 (−0.002 to 1.20)
Multidrug resistance¶	1.83 (1.45–2.25)	1.51 (1.27–1.79)	1.42 (1.16–1.70)		0.32 (−0.17 to 0.82)	0.41 (−0.07 to 0.92)
Amp-only§	1.19 (0.85–1.56)	1.00 (0.78–1.25)	0.96 (0.73–1.21)		0.19 (−0.25 to 0.63)	0.23 (−0.21 to 0.67)
Cef/Amp§	0.49 (0.37–0.65)	0.43 (0.31–0.58)	0.42 (0.30–0.56)		0.06 (−0.13 to 0.26)	0.08 (−0.11 to 0.26)
Cipro§	0.70 (0.55–0.88)	0.29 (0.19–0.41)	0.41 (0.26–0.64)		**0.41 (0.22 to 0.61)‡**	**0.29 (0.02–0.52)‡**

*Amp-only, resistant to ampicillin (MIC >32 µg/mL) but susceptible to ceftriaxone and ciprofloxacin; BHM, Bayesian hierarchical model; Cef/Amp, resistant to ceftriaxone (MIC >4 µg/mL) and ampicillin; Cipro, nonsusceptible to ciprofloxacin (MIC >0.12 µg/mL) but susceptible to ceftriaxone; CrI, credible interval. †Mean estimates of resistance incidence and 95% CrIs were derived using BHMs. Serotypes other than Enteritidis, Typhimurium, Newport, I 4,[*5*],12:i:-, and Heidelberg were combined into the “other” category. For all nontyphoidal *Salmonella*, estimates were derived by summing those for the 6 serotype categories. State-year data were too sparse to use in the BHMs to estimate mean resistance incidence for Cef/Amp among Enteritidis and Cipro among Newport and Heidelberg (4, 5, and 6 Enteritidis isolates, 7, 2, and 8 Newport isolates, and 0, 1, and 3 Heidelberg isolates in 2015–2016, 2004–2008, and 2010–2014, respectively). ‡Resistance incidence in 2015–2016 was compared with that from 2 reference periods, 2004–2008 and 2010–2015 (e.g., increase if 2015–2016 > reference). Mean changes are reported as significant (bold font) if the 95% CrIs (rounded to 2 decimals) do not include 0. §An overall category of clinically important resistance includes any of 3 resistance patterns (i.e., resistant to ceftriaxone, resistant to ampicillin, or nonsusceptible to ciprofloxacin). Amp-only, Cef/Amp, and Cipro are mutually exclusive categories of clinically important resistance. Isolates with any clinically important resistance might have resistance to other agents tested. Model estimates for overall clinically important resistance were derived separately and might differ from the sum of estimates for the 3 mutually exclusive categories. ¶Resistant to >3 classes of antimicrobial agents.

### Changes in Resistance Incidence, 2015–2016 versus Reference Periods

**Figure 2:**
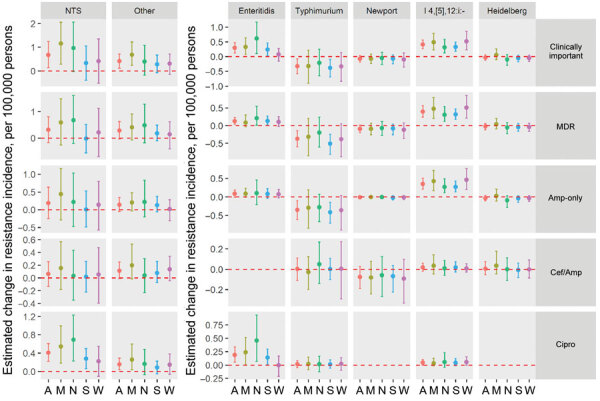
Estimated changes in the incidence of resistant culture-confirmed nontyphoidal *Salmonella* infections, by serotype, resistance category, and geographic region, United States, 2015–2016 versus 2004–2008. Estimated changes in resistance incidence (mean and 95% credible intervals of the posterior differences per 100,000 persons/year) were derived using Bayesian hierarchical models Amp-only, Cef/Amp, and Cipro are mutually exclusive categories of clinically important resistance: Amp-only, resistant to ampicillin but susceptible to ceftriaxone and ciprofloxacin; Cef/Amp, resistant to ceftriaxone and ampicillin; Cipro, nonsusceptible to ciprofloxacin but susceptible to ceftriaxone. Isolates in each category might have resistance to other agents. Multidrug resistance was defined as resistance to >3 classes of antimicrobial agents. The “other” category comprised serotypes other than Enteritidis, Typhimurium, Newport, I 4,[*5*],12:i:-, and Heidelberg. US Census regions were used to define 4 geographic regions (A, all regions; M, Midwest; N, Northeast; S, South; W, West). MDR, multidrug resistant. NTS, all nontyphoidal *Salmonella* serotypes.

**Figure 3:**
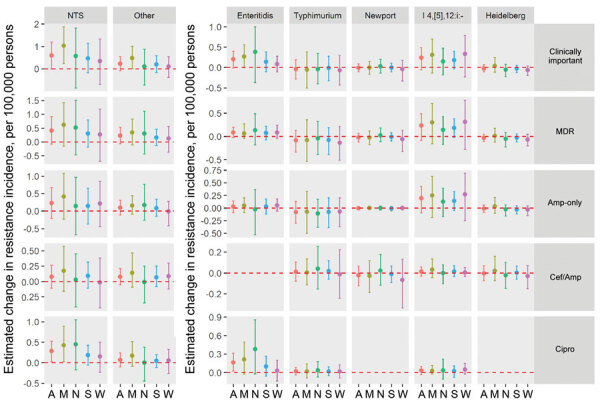
Estimated changes in the incidence of resistant culture-confirmed nontyphoidal *Salmonella* infections, by serotype, resistance category, and geographic region, United States, 2015–2016 versus 2010–2014. Estimated changes in resistance incidence (mean and 95% credible intervals of the posterior differences per 100,000 persons/year) were derived using Bayesian hierarchical models. Amp-only, Cef/Amp, and Cipro are mutually exclusive categories of clinically important resistance: Amp-only, resistant to ampicillin but susceptible to ceftriaxone and ciprofloxacin; Cef/Amp, resistant to ceftriaxone and ampicillin; Cipro, nonsusceptible to ciprofloxacin but susceptible to ceftriaxone. Isolates in each category might have resistance to other agents. Multidrug resistance (MDR) was defined as resistance to >3 classes of antimicrobial agents. The “other” category comprised serotypes other than Enteritidis, Typhimurium, Newport, I 4,[*5*],12:i:-, and Heidelberg. US Census regions were used to define 4 geographic regions (A, all regions; M, Midwest; N, Northeast; S, South; W, West). MDR, multidrug resistant; NTS, all nontyphoidal *Salmonella* serotypes.

The mean annual incidence of infections with any clinically important resistance increased during 2015–2016 compared with 2004–2008; there was no significant change compared with 2010–2014 (Table 2; Figures 2 and 3). Among the resistance categories, the mean annual incidence of ciprofloxacin-nonsusceptible *Salmonella* infections increased during 2015–2016 compared with both reference periods.

### Changes in Resistance Incidence, 2015–2016 versus 2004–2008

The mean annual incidence of *Salmonella* infections with clinically important resistance increased by 0.68 (95% CrI 0.13–1.24)/100,000 persons (Table 2). By census region, a significant increase in resistance only occurred in the Midwest (Figure 2). By serotype, I 4,[*5*],12:i:- had an incidence increase of 0.41(95% CrI 0.27–0.56)/100,000 persons, accounting for 37% of the increase in clinically important resistant *Salmonella* infections (Appendix Table 2). The incidence of resistant I 4,[*5*],12:i:- infections increased significantly in all 4 regions, with highest increase in the West and Midwest. Enteritidis infections with clinically important resistance increased by 0.29 (95% CrI 0.12–0.47)/100,000 persons, accounting for 26% of the increase in resistant infections. This increase was significant in 3 regions, with highest increase in the Northeast. Infections with clinically important resistance caused by serotypes categorized as other increased by 0.41 (95% CrI 0.12–0.72)/100,000 persons, accounting for 37% of the increase in resistant infections (Figure 2; Appendix Table 2). Typhimurium infections with clinically important resistance decreased (−0.33 [95% CrI –0.58 to −0.07]/100,000 persons).

Although no significant changes were noted in the mean annual incidence of *Salmonella* infections with multidrug or ampicillin-only resistance, some serotypes did change (Figure 2; Appendix Table 2). MDR I 4,[*5*],12:i:- infections increased (0.40 [95% CrI 0.24–0.56]/100,000 persons); this change was significant in all 4 regions, with highest increase in the West and Midwest. The incidence of MDR Enteritidis infections also increased (0.13 [95% CrI 0.04–0.23]/100,000 persons). We observed a decrease in Typhimurium infections with multidrug resistance (−0.37 [95% CrI –0.59 to −0.14]/100,000 persons) and ampicillin-only resistance (−0.35 [95% CrI –0.61 to −0.10]/100,000 persons). Serotype I 4,[*5*],12:i:- infections with ampicillin-only resistance increased (0.35 [95% CrI 0.21–0.50]/100,000 persons); this change was significant in all 4 regions, with highest increase in the West and Midwest.

The mean annual incidence of ciprofloxacin-nonsusceptible *Salmonella* infections increased by 0.41 (95% CrI 0.22–0.61)/100,000 persons (Table 2). Ciprofloxacin-nonsusceptible Enteritidis infections increased by 0.19 (95% CrI 0.05–0.34)/100,000 persons, accounting for 47% of the increase in these infections (Appendix Table 2). This increase was significant in 3 regions, most notably in the Northeast (Figure 2). Ciprofloxacin-nonsusceptible infections caused by serotypes categorized as other increased by 0.16 (95% CrI 0.04–0.29)/100,000 persons, accounting for 38% of the increase in ciprofloxacin-nonsusceptible infections (Figure 2; Appendix Table 2).

### Changes in Resistance Incidence, 2015–2016 versus 2010–2014

The mean annual incidence of *Salmonella* infections with clinically important resistance did not change compared with the previous 5 years. However, the mean annual incidence of ciprofloxacin-nonsusceptible *Salmonella* infections increased by 0.29 (95% CrI 0.02–0.52)/100,000 persons (Table 2); by region, the increase was significant only in the Midwest (Figure 3). Ciprofloxacin-nonsusceptible Enteritidis infections increased by 0.16 (95% CrI 0.02–0.32)/100,000 persons, accounting for 57% of the increase in ciprofloxacin-nonsusceptible infections (Appendix Table 3).

### Extrapolation to the US population

Compared with the number of infections for 2004–2008, an estimated ≈63,000 more *Salmonella* infections with clinically important resistance occurred each year during 2015–2016, from an average of ≈159,000 to ≈222,000; more than half were ciprofloxacin-nonsusceptible (Table 3). Compared with the number of infections for previous 5 years, an estimated ≈56,000 more *Salmonella* infections with clinically important resistance occurred each year during 2015–2016; more than half were ciprofloxacin-nonsusceptible.

**Table 3:** Point estimates of the total number and changes in the total number of resistant nontyphoidal *Salmonella* infections extrapolated to the US population, by resistance category, United States, 2015–2016 versus 2004–2008 and 2010–2014*†

Resistance category					Change in no. infections/year‡	
	No. infections/year†				2015–2016 vs. 2004–2008	2015–2016 vs. 2010–2014
	2015–2016	2004–2008	2010–2014			
Any clinically important resistance§	222,000	159,000	166,000		**63,000‡**	56,000
Multidrug resistance¶	171,000	141,000	133,000		30,000	38,000
Amp-only§	111,000	93,000	90,000		18,000	21,000
Cef/Amp§	46,000	40,000	39,000		6,000	7,000
Cipro§	65,000	27,000	38,000		**38,000‡**	**27,000‡**

*Amp-only, resistant to ampicillin (MIC >32 µg/mL) but susceptible to ceftriaxone and ciprofloxacin; BHM, Bayesian hierarchical model; Cef/Amp, resistant to ceftriaxone (MIC >4 µg/mL) and ampicillin; Cipro, nonsusceptible to ciprofloxacin (MIC >0.12 µg/mL) but susceptible to ceftriaxone; CrI, credible interval. †Point estimates extrapolated to the entire US population were calculated by multiplying mean estimates for culture-confirmed infections (derived using BHM) by the multiplier of 29 and the average total U.S. population for 2015–2016 (322 million). The multiplier of 29 is the mean estimate of the total number of infections for every culture-confirmed nontyphoidal *Salmonella* infection. The 95% CrIs were not derived. Extrapolated point estimates were rounded to the nearest thousand. ‡Model-derived mean estimates of changes in resistance incidence (Table 2) used to calculate extrapolated estimates are reported as significant if the 95% CrIs do not include 0; the extrapolated estimates corresponding to these BHM-derived estimates are shown in bold font. Although the 95% CrIs of extrapolated estimates were not derived, they can be assumed to include 0 if the 95% CrIs of BHM-derived estimates include 0. §An overall category of clinically important resistance includes any of 3 resistance patterns (i.e., resistant to ceftriaxone, resistant to ampicillin, or nonsusceptible to ciprofloxacin). Amp-only, Cef/Amp, and Cipro are mutually exclusive categories of clinically important resistance. Isolates with any clinically important resistance might have resistance to other agents tested. Model estimates for overall clinically important resistance were derived separately and might differ from the sum of BHM estimates for the 3 mutually exclusive categories; thus, extrapolated estimates for the overall category might differ from the sum of mutually exclusive categories. ¶Resistant to >3 classes of antimicrobial agents.

## Discussion

Our analysis indicates that the incidence of resistant *Salmonella* infections was higher in 2015–2016 than in earlier periods during 2004–2014. The annual incidence of culture-confirmed infections with clinically important resistance increased by 0.68/100,000 persons, a 40% increase in the annual number of infections, during 2015–2016 compared with 2004–2008. Serotypes I 4,[5],12:i:- and Enteritidis were responsible for two thirds of this increase. Ciprofloxacin-nonsusceptible infections accounted for more than half of the increase. Extrapolating to total infections in the US population using a multiplier to account for unreported infections resulted in an estimated ≈63,000 more infections with clinically important resistance per year during 2015–2016 compared with 2004–2008 (from ≈159,000 to ≈222,000 infections).

The increased incidence of ciprofloxacin-nonsusceptible *Salmonella* infections during 2015–2016 compared with incidence for both 2004–2008 and 2010–2014 is a concerning trend. Serotype Enteritidis contributed the most to this increase. Although the incidence of infections with Enteritidis, the most common serotype, has not changed significantly in >10 years, the percentage of ciprofloxacin-nonsusceptible infections has increased almost steadily (*11*,*16*). Chicken and eggs have been the main domestic sources of Enteritidis infections (*29*,*30*). About 20% of Enteritidis infections are linked to international travel, which is an important source of ciprofloxacin-nonsusceptible Enteritidis infections (*8*,*31*,*32*).

The incidence of infections with clinically important resistance and ciprofloxacin-nonsusceptibility caused by serotypes categorized as other was higher during 2015–2016 than during 2004–2008. Some of these serotypes are emerging or have concerning levels of resistance, including Dublin, Infantis, Kentucky, Hadar, and Agona (*16*,*24*,*33*). Some have been associated with resistance, invasive illness, or both (*11*,*19*,*23*,*33*).

The decrease in resistant Typhimurium infections might be related to the simultaneous increase in I 4,[5],12:i- infections, which some call monophasic Typhimurium (*16*,*18*,*34*). In the 1990s, MDR Typhimurium infections increased markedly in Europe and then in the United States (*35*,*36*). Most isolates from these infections that underwent phage typing were definitive type 104 (*14*,*21*,*35*,*36*). Isolations of this strain have decreased globally; the reasons are not known (*36*)

Changes in resistance incidence by resistance category and serotype varied by geographic region, with significant increases in most regions for serotypes I 4,[*5*],12:i:- and Enteritidis. An increase in the incidence of I 4,[*5*],12:i:- infections with multidrug and ampicillin-only resistance occurred in all 4 regions, with highest increase in the West and Midwest. Pork products have been associated with I 4,[*5*],12:i:- infections with resistance to ampicillin, sulfonamide, streptomycin, and tetracycline in the West (*34*,*37*). The regional pattern of pork consumption has reflected the regional pattern of pork production, which is highest in the Midwest; 8 of the 10 states with the highest production of swine are in the Midwest (*38*,*39*). A study showed that MDR I 4,[*5*],12:i:- strains from swine in the Midwest during 2014–2016 were typically resistant to ampicillin, sulfonamide, streptomycin, and tetracycline and probably part of a European clade that has spread in the United States and elsewhere; these strains harbored plasmid-mediated resistance genes, which can be transmitted horizontally to other bacteria (*34*). This trend could partly explain the widespread increase in the incidence of MDR I 4,[*5*],12:i:- infections. International travel could have contributed to an increase in the incidence of ciprofloxacin-nonsusceptible Enteritidis infections, which increased in 3 regions and was highest in the Northeast. International travel has increased since 2014, and residents of northeastern states accounted for more than one third of US travelers during 2015–2016 (*40*). In the United Kingdom, an increase in these infections has been linked to international travel and imported foods (*41*). In the United States, ciprofloxacin-nonsusceptible strains of Enteritidis and other serotypes have been isolated from imported seafood (*42*). Plasmid-mediated quinolone-resistance genes have been detected among ciprofloxacin-nonsusceptible isolates in the United States; these genes might contribute to spread of fluoroquinolone nonsusceptibility (*43*).

Our use of a Bayesian hierarchical model improved the estimates, as shown by the smoothing of resistance incidence and temporal change lines, by addressing issues related to missing and sparse state-year data (*20*,*26*). Our method of calculating the average difference in incidence between groups of years is more refined than approaches using a negative binomial model because it does not assume homogeneous resistance incidence rates across multiple years (*11*,*44*). It is therefore less likely to underestimate the variability of estimated changes. However, this analysis is subject to the same limitations described in previous reports, including unmeasured sources of bias and uncertainty derived by combining data from separate unlinked surveillance systems (*20*,*26*). Our estimates of significant changes were limited to comparisons with the reference periods used to assess changes in resistance percentages in NARMS annual reports (*16*). Our choice to compare a recent 2-year period with earlier 5-year periods balanced the need to assess the most current situation with the need for sufficient data to assess significant changes. Because of the low percentage of isolates showing resistance to trimethoprim/sulfamethoxazole (<3%) or decreased susceptibility to azithromycin (<1%), an important agent used to treat serious infections, we did not provide estimates for these agents (*2**,**16**,**18**,**20*). We included infections resistant to ceftriaxone and nonsusceptible to ciprofloxacin in the ceftriaxone/ampicillin-resistance category; they represented only 0.3% of *Salmonella* isolates submitted to NARMS. The fact that some ciprofloxacin nonsusceptible infections were not included in the ciprofloxacin nonsusceptible category further supports our finding that ciprofloxacin-nonsusceptible infections increased during the study period. Increasing use of culture-independent diagnostic tests by clinical laboratories can change the submission of isolates to public health laboratories and reporting of infections (*11*); these changes warrant adjustments in future analyses (*20*).

We multiplied estimates of culture-confirmed infections by 29 to account for undiagnosed infections. However, resistant infections are associated with more severe illness, so they might be more likely to be detected (*3*–*6*). Thus, the appropriate multiplier (the ratio of total infections to culture-confirmed infections) for resistant infections might be <29. To calculate undiagnosed *Salmonella* infections, multipliers of 12 for persons <5 years of age and 23 for persons > 65 years of age have been reported (*45*). Although children <5 years of age have the highest incidence of *Salmonella* infections, older adults might disproportionately account for resistant infections because they are more likely to have serious illness and be hospitalized (*4**,**5**,**44**–**47*); therefore, a multiplier of 23 might be an appropriate choice. However, we chose 29 because it was used in a previous estimate of the total number of *Salmonella* infections in the population (*1*) and because persons 5–64 years of age account for most culture-confirmed infections reported to CDC and most isolates with clinically important resistance submitted to NARMS (*4**,**18**,**44**,**45*). We did not attach uncertainties to the extrapolated total number of resistant infections and changes in that number because uncertainties of the multiplier are not known. Although resistance incidence can vary by demographic subgroup, geographic region, time, and other factors, we did not include additional uncertainties from the extrapolation to the US population using the average 2015–2016 population estimates for the 50 states (*19*,*21*,*22*,*46*,*47*).

Estimates of changes in resistance incidence can help identify trends of greatest concern to set priorities for prevention. Analyses that include the varying distributions of infections by demographic subgroups, season, and recent travel could inform serotype-specific, regional, and source-targeted prevention strategies (*5*,*11*,*21*,*22*,*31*,*44*–*48*). The increasing use of whole-genome sequencing by public health laboratories to characterize *Salmonella* strains will enhance surveillance of antimicrobial-resistant *Salmonella* from human and nonhuman sources (*49*). Antimicrobial agents contribute to resistance wherever they are used, including in food animals and humans (*50*). A One Health approach can help in detecting and controlling antimicrobial resistance, which is a complex and multifaceted problem that affects humans, animals, and the environment (*50*).

Appendix Additional information about increased incidence of antimicrobial-resistant nontyphoidal *Salmonella* infections, United States, 2004–2016.
