# Supplementary material for: Increased Incidence of Antimicrobial-Resistant Nontyphoidal Salmonella Infections, United States, 2004–2016
Source: Emerg Infect Dis. 2021 Jun;27(6):1662–72. doi: 10.3201/eid2706.204486 (PMC8153855; doi:10.3201/eid2706.204486)
Supplement: Appendix — Additional information about increased incidence of antimicrobial-resistant nontyphoidal Salmonella infections, United States, 2004–2016. [file 20-4486-Techapp-s1.pdf]

Article DOI: https://doi.org/10.3201/eid2706.204486

Increased Incidence of Antimicrobial-Resistant Nontyphoidal *Salmonella* Infections, United States, 2004–2016

Appendix

Appendix Table 1. Population estimates, number of isolates reported and tested, and percentage of isolates with clinically important and multidrug resistance, by year, United States, 2004–2016*

Year	Population†	No. reported to LEDS‡	No. tested in NARMS‡	Isolates with clinically important resistance§ No. (%)	Isolates with multidrug resistance¶ No. (%)
2004	290,304,689	34,374	1,762	245 (13.9)	200 (11.4)
2005	292,989,788	35,116	2,016	258 (12.8)	238 (11.8)
2006	295,824,198	38,737	2,151	281 (13.1)	254 (11.8)
2007	298,660,828	37,840	2,115	253 (12.0)	232 (11.0)
2008	301,494,062	43,505	2,306	274 (11.9)	223 (9.7)
2009	304,133,689	38,669	2,136	241 (11.3)	208 (9.7)
2010	306,665,034	43,397	2,418	272 (11.2)	225 (9.3)
2011	308,942,304	42,916	2,305	255 (11.1)	212 (9.2)
2012	311,240,143	44,824	2,201	241 (10.9)	192 (8.7)
2013	313,412,383	41,885	2,157	277 (12.8)	214 (9.9)
2014	315,751,397	44,234	2,093	259 (12.4)	194 (9.3)
2015	318,063,375	47,811	2,322	375 (16.1)	286 (12.3)
2016	320,275,892	46,554	2283	315 (13.8)	234 (10.2)
Total		539,862	28,265	3,546 (12.5)	2,912 (10.3)

*LEDS, Laboratory-based Enteric Diseases Surveillance; NARMS, National Antimicrobial Resistance Monitoring System.
†Total estimates from the U.S. Census Bureau for 48 states (Alaska, Hawaii, and District of Columbia excluded).
‡Number of infections reported by the 48 contiguous states to LEDS and number of isolates submitted by the 48 states and tested in NARMS.
§Overall category includes any of 3 clinically important resistance patterns (i.e., resistant to ceftriaxone, resistant to ampicillin, or nonsusceptible to ciprofloxacin).
¶Resistant to $\geq$3 classes of antimicrobial agents.

Appendix Table 2. Estimated changes in the incidence of resistant culture-confirmed nontyphoidal *Salmonella* infections, by serotype and resistance category: 2015–2016 versus 2004–2008*

Resistance category	Type of change†	Mean change†‡ in resistance incidence (per 100,000 persons/year) 2015–2016 vs. 2004–2008 (% contribution of serotype)						Total increase/ decrease	All NTS† (net change)
		Enteritidis	Typhimurium	Newport	I 4,[5],12:i:-	Heidelberg	Other†		
Any clinically important resistance§	↑	**0.29‡** (26)	–	–	**0.41‡** (37)	–	**0.41‡** (37)	1.11	**0.68‡**
	↓	–	**-0.33‡** (75)	−0.07 (17)	–	-0.04 (8)	–	−0.44	–
Multidrug resistance¶	↑	**0.13‡** (16)	–	–	**0.40‡** (49)	–	0.28 (35)	0.81	0.32
	↓	–	**-0.37‡** (76)	−0.09 (19)	–	−0.03 (5)	–	−0.49	–
Amp-only*§	↑	0.08 (15)	–	–	**0.35‡** (61)	–	0.14 (24)	0.57	0.19
	↓	–	**-0.35‡** (89)	−0.01 (3)	–	−0.03 (8)	–	−0.39	–
Cef/Amp*§	↑	NC†	0.003 (2)	–	0.02 (13)	0.01 (4)	0.11 (81)	0.14	0.06
	↓	NC†	–	−0.08 (100)	–	–	–	−0.08	–
Cipro*§	↑	**0.19‡** (47)	0.02 (4)	NC†	0.04 (11)	NC†	**0.16‡** (38)	0.41	**0.41‡**

*Amp-only, resistant to ampicillin but susceptible to ceftriaxone and ciprofloxacin; Cef/Amp, resistant to ceftriaxone and ampicillin; Cipro, nonsusceptible to ciprofloxacin but susceptible to ceftriaxone; CrI, credible interval; NC, not calculated; NTS, nontyphoidal *Salmonella*; ↑, increase; ↓, decrease.

†Mean estimates and 95% CrIs for each resistance and serotype category were derived using Bayesian hierarchical models. Resistance incidence in 2015–2016 was compared with that for 2004–2008 (↑ if 2015–2016 >2004–2008, ↓ if 2015–2016 <2004–2008). Serotypes other than Enteritidis, Typhimurium, Newport, I 4,[5],12:i:-, and Heidelberg were combined in the "other" category. For all NTS, estimated changes were derived by summing those for the 6 serotype categories (net increase or decrease). State-year data were too sparse to use in the Bayesian hierarchical models to estimate resistance incidence for Cef/Amp among Enteritidis and for Cipro among Newport and Heidelberg; thus, estimated changes in resistance incidence were not calculated (NC).

‡Mean changes are reported as significant (bold font) if the 95% CrIs (rounded to 2 decimals) do not include 0: any clinically important resistance, Enteritidis (0.29 [95% CrI 0.12, 0.47]), I 4,[5],12:i:- (0.41 [0.27, 0.56]), Typhimurium (−0.33 [-0.58, −0.07]), Other (0.41 [0.12, 0.72]); MDR, Enteritidis (0.13 [0.04, 0.23]), I,4,[5],12:i:- (0.40 [0.24, 0.56]), Typhimurium (−0.37 [-0.59, −0.14]); Amp-only, I 4,[5],12:i:- (0.35 [0.21, 0.50]), Typhimurium (−0.35 [-0.61, −0.10]); Cipro, Enteritidis (0.19 [0.05, 0.34]), Other (0.16 [0.04, 0.29]);

§An overall category of clinically important resistance includes any of 3 resistance patterns (i.e., resistant to ceftriaxone, resistant to ampicillin, or nonsusceptible to ciprofloxacin). Amp-only, Cef/Amp, and Cipro are mutually exclusive categories of clinically important resistance

¶Resistant to ≥3 classes of antimicrobial agents.

Appendix Table 3. Estimated changes in the incidence of resistant culture-confirmed nontyphoidal *Salmonella* infections, by serotype and resistance category: 2015–2016 versus 2010–2014*

Resistance category	Type of change†	Mean change†‡ in resistance incidence (per 100,000 persons/year) 2015–2016 vs. 2010–2014 (% contribution of serotype)						Total increase/ decrease	All NTS† (net change)
		Enteritidis	Typhimurium	Newport	I 4,[5],12:i:-	Heidelberg	Other†		
Any clinically important resistance§	↑	0.20 (30)	–	–	0.24 (36)	–	0.23 (34)	0.67	0.60
	↓	–	−0.04 (56)	−0.01 (9)	–	−0.03 (35)`	–	−0.07	–
Multidrug resistance¶	↑	0.09 (16)	–	–	0.24 (43)	–	0.22 (41)	0.55	0.41
	↓	–	−0.08 (64)	−0.02 (13)	–	−0.03 (23)	–	−0.13	–
Amp-only*§	↑	0.03 (9)	–	–	0.20 (61)	–	0.10 (30)	0.32	0.23
	↓	–	−0.08 (85)	−0.002 (2)	–	−0.01 (13)	–	−0.09	–
Cef/Amp*§	↑	NC†	0.01 (13)	–	0.01 (13)	–	0.07 (74)`	0.10	0.08
	↓	NC†	–	−0.02 (83)	–	−0.004 (17)`	–	−0.03	
Cipro*§	↑	**0.16‡** (57)	0.02 (7)	NC†	0.03 (11)	NC†	0.07 (25)`	0.29	**0.29‡**

*Amp-only, resistant to ampicillin but susceptible to ceftriaxone and ciprofloxacin; Cef/Amp, resistant to ceftriaxone and ampicillin; Cipro, nonsusceptible to ciprofloxacin but susceptible to ceftriaxone; CrI, credible interval; NC, not calculated; NTS, nontyphoidal *Salmonella*; ↑, increase; ↓, decrease.

†Mean estimates and 95% credible intervals (CIs) for each resistance and serotype category were derived using Bayesian hierarchical models. Resistance incidence in 2015–2016 was compared with that for 2010–2014 (↑ if 2015–2016 >2010–2014, ↓ if 2015–2016 <2010–2014). Serotypes other than Typhimurium, Enteritidis, I 4,[5],12:i:-, Newport, and Heidelberg were combined in the "other" category. For all NTS, estimated changes were derived by summing those for the 6 serotype categories (net increase or decrease). State-year data were too sparse to use in the Bayesian hierarchical models to estimate resistance incidence for Cef/Amp among Enteritidis and for Cipro among Newport and Heidelberg; thus, estimated changes in resistance incidence were not calculated (NC).

‡Mean change is reported as significant (bold font) if the 95% CIs (rounded to 2 decimals) do not include 0: Cipro, Enteritidis (0.16 [95% CrI 0.02. 0.32]).

§An overall category of clinically important resistance includes any of 3 resistance patterns (i.e., resistant to ceftriaxone, resistant to ampicillin, nonsusceptible to ciprofloxacin). Amp-only, Cef/Amp, and Cipro are mutually exclusive categories of clinically important resistance.

¶Resistant to ≥3 classes of antimicrobial agents.

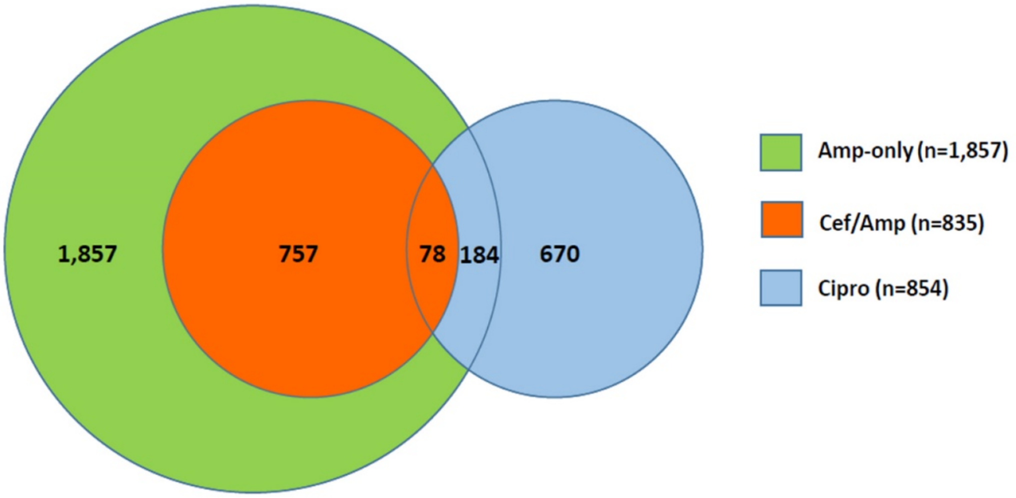

Appendix Figure 1. Number of nontyphoidal *Salmonella* isolates with clinically important resistance, by mutually exclusive resistance category, 2004–2016. Three mutually exclusive categories of clinically important resistance were defined: Amp-only as resistant to ampicillin but susceptible to ceftriaxone and ciprofloxacin; Cef/Amp as resistant to ceftriaxone and ampicillin; and Cipro as nonsusceptible to ciprofloxacin but susceptible to ceftriaxone. Isolates in each category may have resistance to other agents.

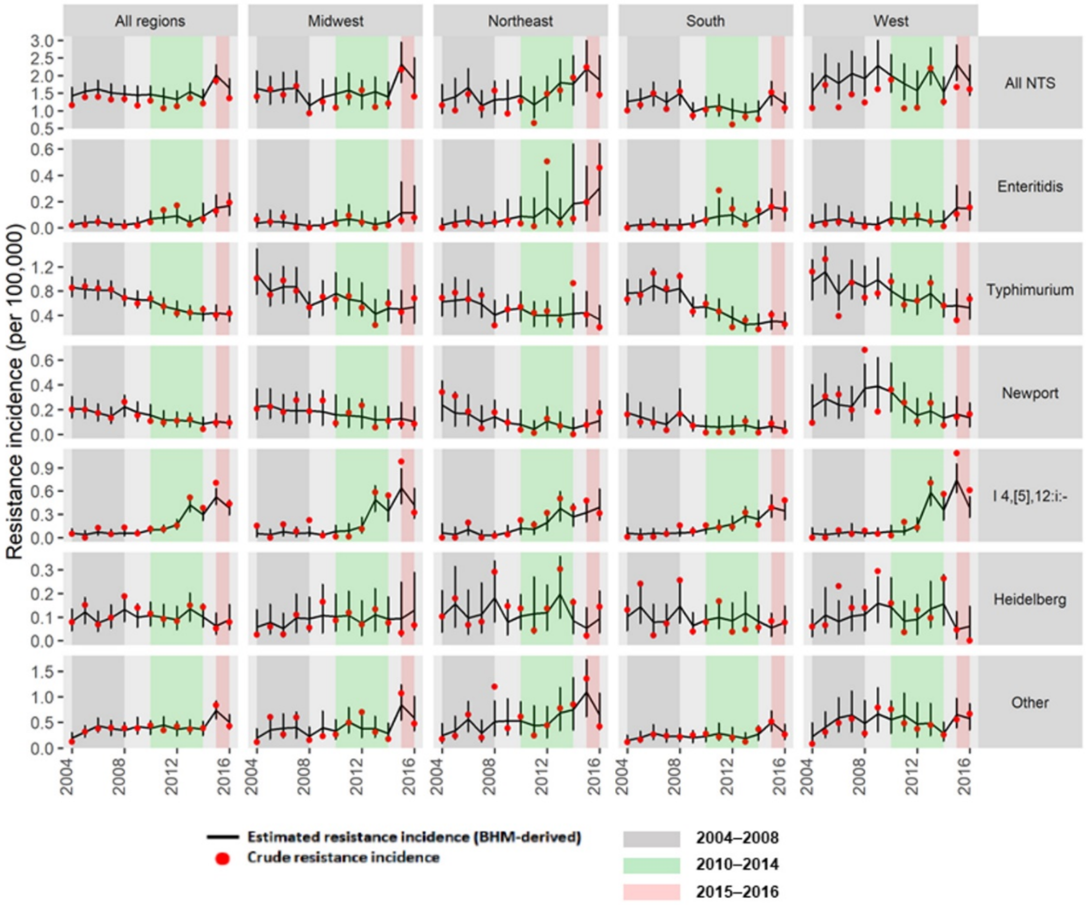

Appendix Figure 2. Estimated annual incidence of culture-confirmed nontyphoidal *Salmonella* infections with multidrug resistance, by serotype and region, 2004–2016. Estimated changes in resistance incidence (mean and 95% credible intervals of the posterior differences per 100,000 persons/year) were derived using Bayesian hierarchical models (BHM). Crude resistance incidence rates were derived by multiplying infection incidence and resistance proportion for state-year. Multidrug resistance (MDR) was defined as resistance to three or more classes of antimicrobial agents. The “other” category comprised serotypes other than Enteritidis, Typhimurium, Newport, I 4,[5],12:i:-, and Heidelberg. U.S. Census regions were used to define 4 geographic regions. NTS, all nontyphoidal *Salmonella* serotypes.

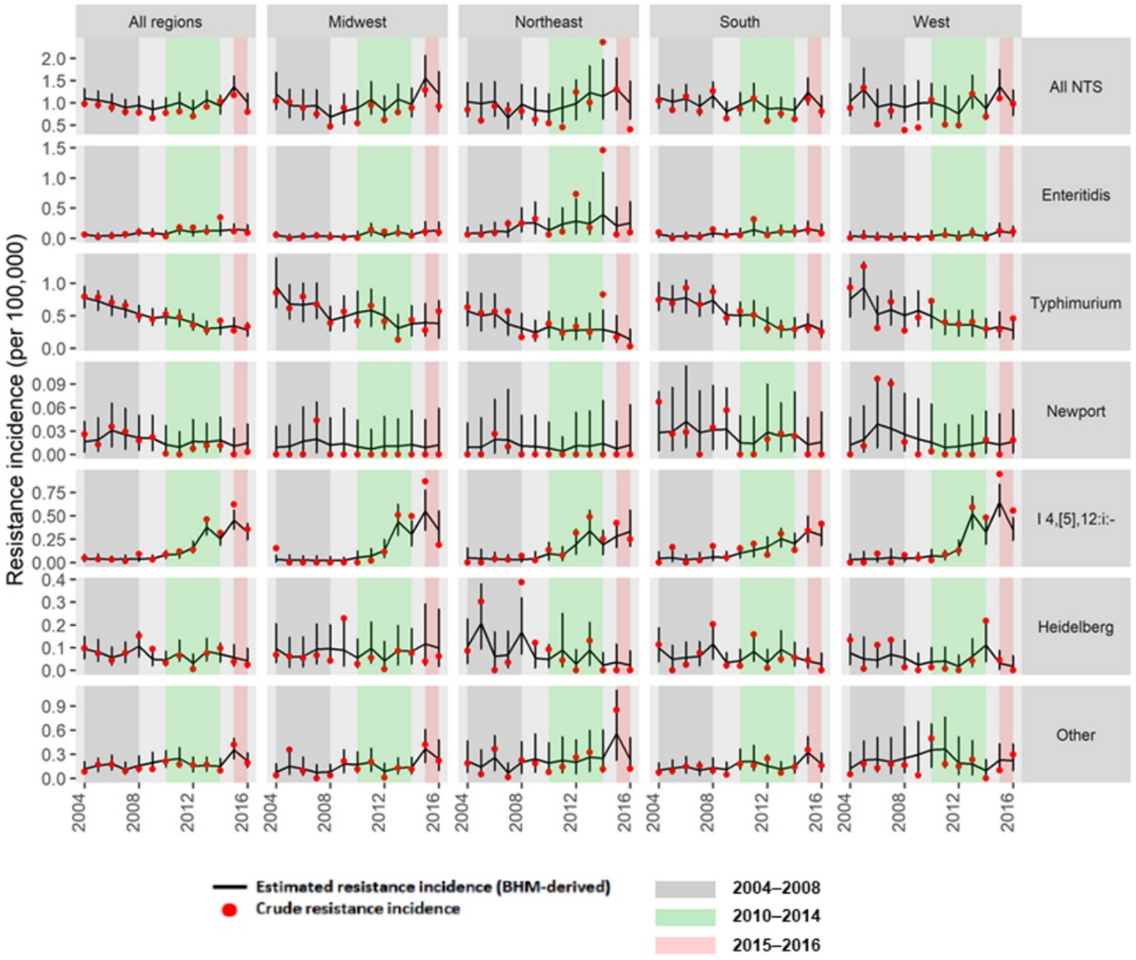

Appendix Figure 3. Estimated annual incidence of culture-confirmed nontyphoidal *Salmonella* infections with ampicillin-only resistance (Amp-only), by serotype and region, 2004–2016. Estimated changes in resistance incidence (mean and 95% credible intervals of the posterior differences per 100,000 persons/year) were derived using Bayesian hierarchical models (BHM). Crude resistance incidence rates were derived by multiplying infection incidence and resistance proportion for state-year. Amp-only was defined as resistant to ampicillin but susceptible to ceftriaxone and ciprofloxacin. The “other” category comprised serotypes other than Enteritidis, Typhimurium, Newport, I 4,[5],12:i:-, and Heidelberg. U.S. Census regions were used to define 4 geographic regions. NTS, all nontyphoidal *Salmonella* serotypes.

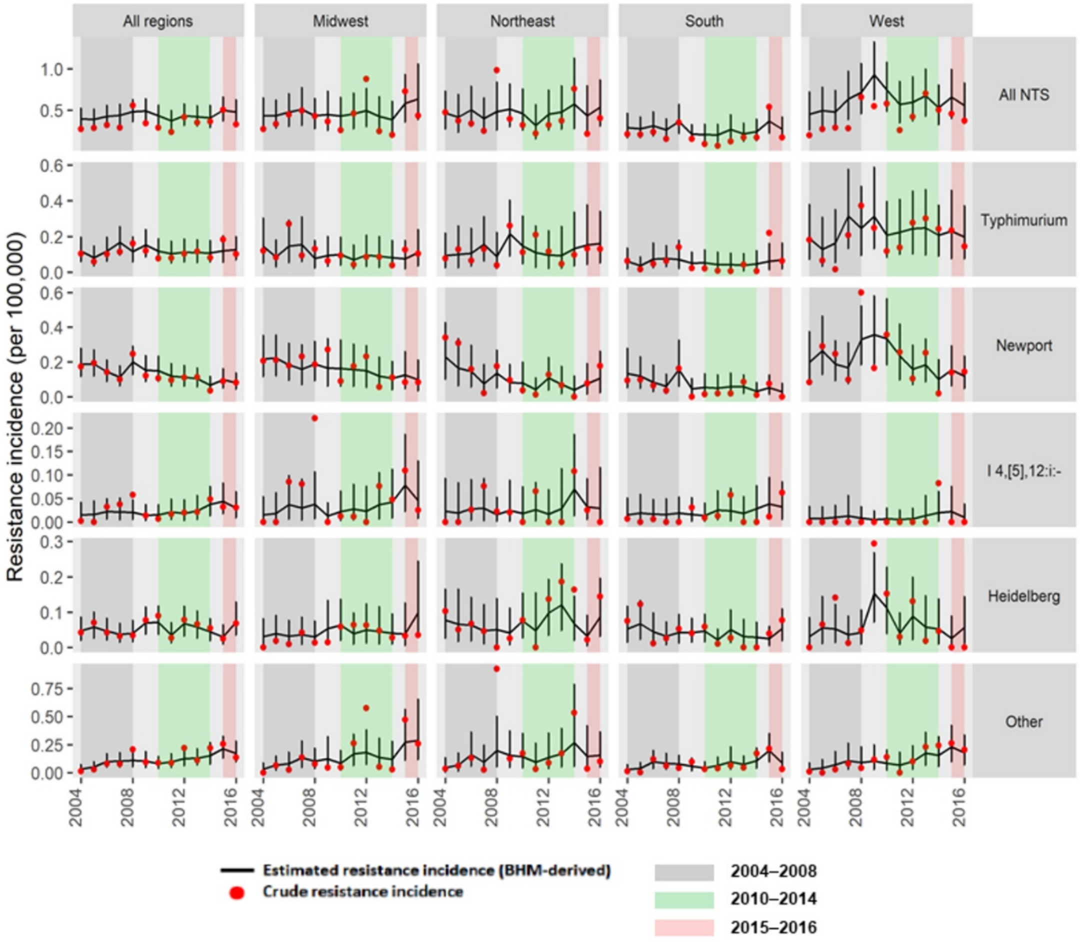

Appendix Figure 4. Estimated annual incidence of culture-confirmed nontyphoidal *Salmonella* infections with ceftriaxone/ampicillin resistance (Cef/Amp), by serotype and region, 2004–2016. Estimated changes in resistance incidence (mean and 95% credible intervals of the posterior differences per 100,000 persons/year) were derived using Bayesian hierarchical models (BHM). Crude resistance incidence rates were derived by multiplying infection incidence and resistance proportion for state-year. Cef/Amp was defined as resistant to ceftriaxone and ampicillin. The “other” category comprised serotypes other than Enteritidis, Typhimurium, Newport, I 4,[5],12:i:-, and Heidelberg; estimates for Enteritidis (not included in the figure) were not derived because state-year data were too sparse to use in the BHM. US Census regions were used to define 4 geographic regions. NTS, all nontyphoidal *Salmonella* serotypes.

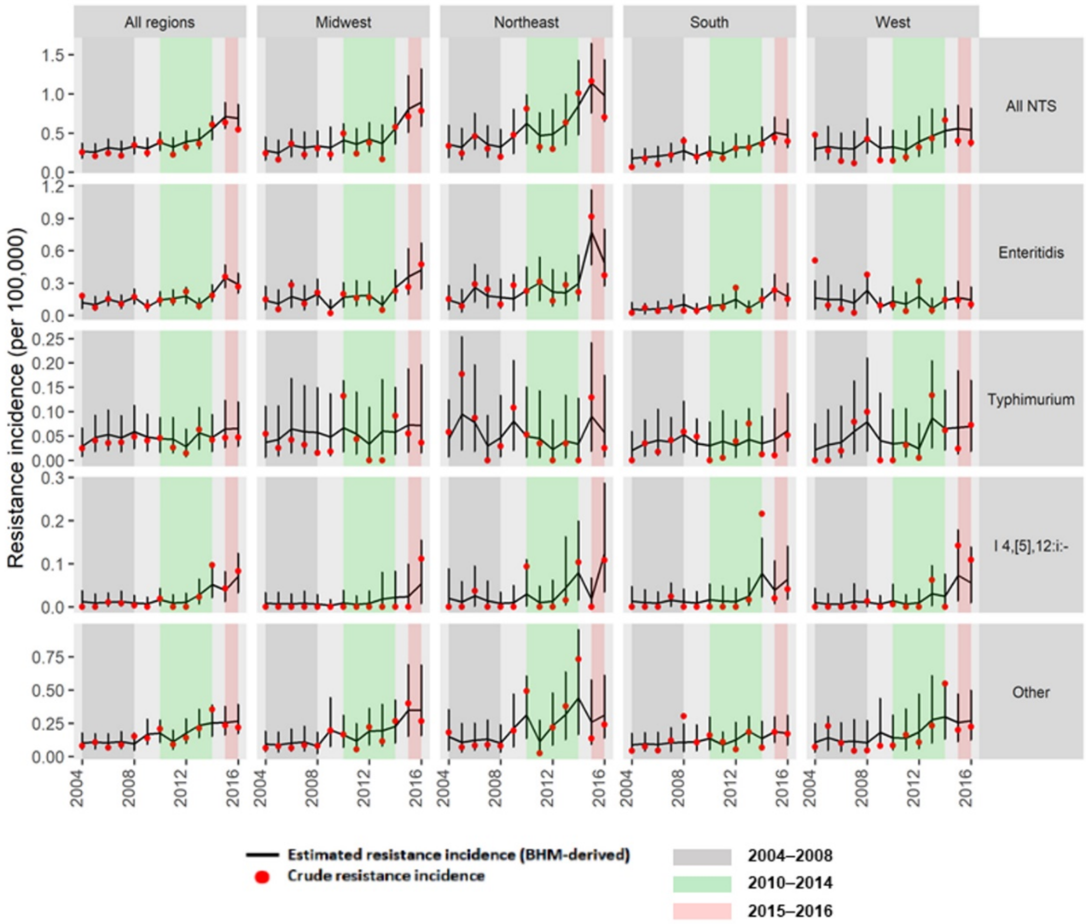

Appendix Figure 5. Estimated annual incidence of culture-confirmed nontyphoidal *Salmonella* infections with ciprofloxacin nonsusceptibility (Cipro), by serotype and region, 2004–2016. Estimated changes in resistance incidence (mean and 95% credible intervals of the posterior differences per 100,000 persons/year) were derived using Bayesian hierarchical models (BHM). Crude resistance incidence rates were derived by multiplying infection incidence and resistance proportion for state-year. Cipro was defined as nonsusceptible to ciprofloxacin but susceptible to ceftriaxone. The “other” category comprised serotypes other than Enteritidis, Typhimurium, Newport, I 4,[5],12:i:-, and Heidelberg; estimates for Newport and Heidelberg (not included in the figure) were not derived because state-year data were too sparse to use in the BHM. US Census regions were used to define 4 geographic regions. NTS, all nontyphoidal *Salmonella* serotypes.

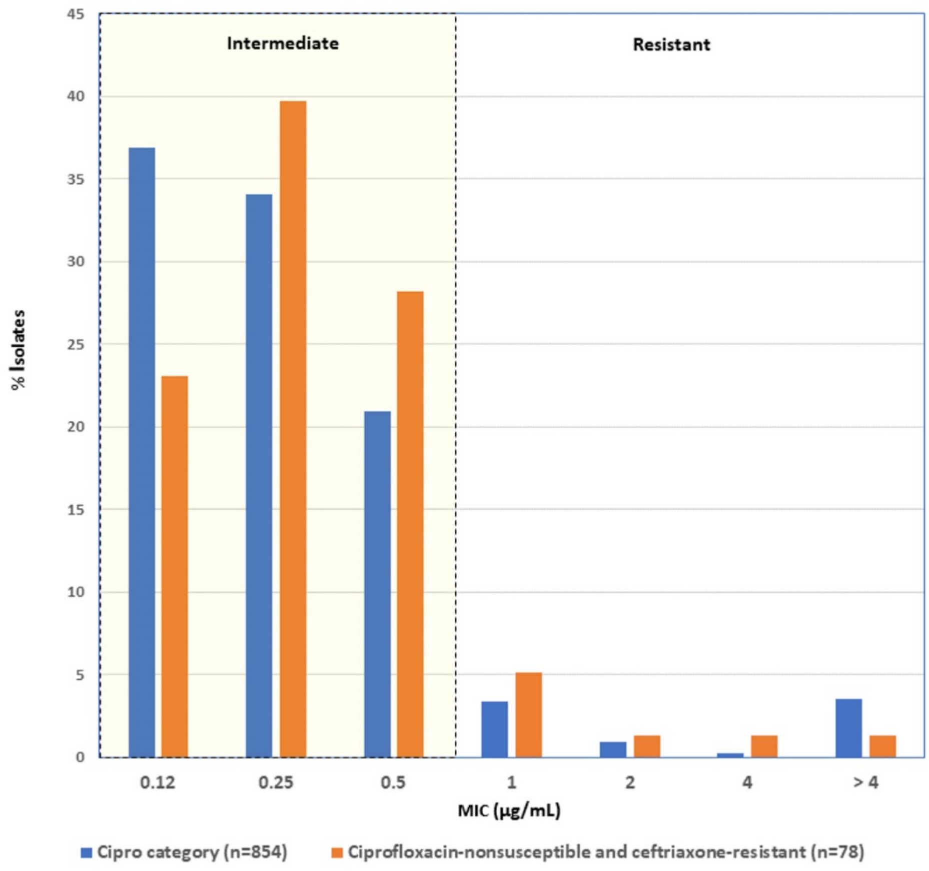

Appendix Figure 6. Distribution of ciprofloxacin MICs among Cipro category (i.e., ciprofloxacin-nonsusceptible and ceftriaxone-susceptible) and ciprofloxacin-nonsusceptible and ceftriaxone-resistant *Salmonella* isolates, 2004–2016. Of 854 isolates in the Cipro category, 785 (92%) had MICs within the intermediate range, 0.12–0.5 µg/mL. Of 78 isolates nonsusceptible to ciprofloxacin and ceftriaxone-resistant, 71 (91%) had MICs within the intermediate range; these 78 isolates were not included in the Cipro category.
